# Trauma-related obsessive–compulsive disorder: a review

**DOI:** 10.1080/21642850.2014.905207

**Published:** 2014-04-23

**Authors:** Kristy L. Dykshoorn

**Affiliations:** ^a^ Department of Educational Psychology, University of Alberta, 6–102 Education North, Edmonton, ABT6G 2G5, Canada

**Keywords:** *:* counselling, evidence-based practice, mental health and disorders, post-traumatic stress disorder, obsessive–compulsive disorder

## Abstract

Obsessive–compulsive disorder (OCD) is a highly researched and conceptualized disorder, and yet it remains one of the most debilitating, widespread, and expensive disorders one can be afflicted with [Real, E., Labad, J., Alonso, P., Segalas, C., Jimenez-Murcia, S., Bueno, B., … Menchon, J. M. (2011). Stressful life events at onset of obsessive–compulsive disorder are associated with a distinct clinical pattern. *Depression and Anxiety*, *28*, 367–376. doi:10.1002/da.20792]. Exposure treatments and cognitive-behavioural therapy (CBT) have been largely accepted as best practice for those with OCD, and yet there are still many who are left with “treatment-resistant OCD” [Rowa, K., Antony, M., & Swinson, R. (2007). Exposure and response prevention. In C. Purdon, M. Antony, & L. J. Summerfeldt (Eds.), *Psychological treatment of obsessive-compulsive disorder: Fundamentals and beyond* (pp. 79–109). Washington, DC: American Psychological Association; Foa, E. B. (2010). Cognitive behavioural therapy of obsessive–compulsive disorder. *Dialogues of Clinical Neuroscience*, *12*, 199–207]. Similarly, exposure treatments and CBT have been accepted as best practice for trauma-related distress (i.e. post-traumatic stress disorder; Foa, E. B., Keane, T. M., Friedman, M. J., & Cohen, J. A. (2009). *Effective treatments for PTSD: Practice guidelines from the international society for traumatic studies* (2nd ed.). New York, NY: The Guilford Press). From a literature review, evidence has been provided that demonstrates a high prevalence rate (30–82%) of OCD among individuals with a traumatic history in comparison to the prevalence rate of the general population (1.1–1.8%; [Cromer, K. R., Schmidt, N. B., & Murphy, D. L. (2006). An investigation of traumatic life events and obsessive–compulsive disorder. *Behaviour Research and Therapy*, *45*, 1683–1691. doi:10.1016/j.brat.2006.08.018; Fontenelle, L. F., Cocchi, L., Harrison, B. J., Shavitt, R. G., do Rosario, M. C., Ferrao, Y. A., … Torres, A. R. (2012). Towards a post-traumatic subtype of obsessive–compulsive disorder. *Journal of Anxiety Disorders*, *26*, 377–383. doi:10.1016/j.janxdis.2011.12.001; Gershuny, B. S., Baer, L., Parker, H., Gentes, E. L., Infield, A. L., & Jenike, M. A. (2008). Trauma and posttraumatic stress disorder in treatment-resistant obsessive–compulsive disorder. *Depression and Anxiety*, *25*, 69–71. doi:10.1002/da.20284]). Evidence was collected for a post-traumatic OCD and treatments of trauma-related OCD were considered. OCD and traumatic histories have a significant enough overlap that trauma should be a consideration when treating an individual with OCD. Given the overlap of the client base with OCD and traumatic histories, as well as the overlap in treatment options for those who experience OCD and trauma-induced symptoms, the author will discuss the importance of assessing for traumatic history in clients with OCD as well as approaching treatment from a dual-focus orientation.

## 1. Theoretical and empirical foundations

The interplay between obsessive–compulsive disorder (OCD) and traumatic experiences has been researched consistently in the anxiety disorder community. Many scholars, dating back to Janet's work in 1903, are aware that trauma can and does impact the development of major psychiatric disorders (de Silva & Marks, 2001). Unfortunately, this knowledge has had little impact on the course of treatments available to those suffering from OCD as a result of traumatic life experiences.

### 1.1. Prevalence

According to the Diagnostic and Statistical Manual of Mental Disorders, fifth edition (DSM-V; American Psychiatric Association [APA], 2013), 1.1–1.8% of the world population experience a 12-month prevalence of OCD. Females experience slightly higher rates in adulthood, while males have slightly elevated rates in childhood. Additionally, the 12-month prevalence rate for post-traumatic stress disorder (PTSD) is approximately 3.5% (APA, 2013). This, however, does not include individuals who have experienced trauma-related distress, but do not meet the criteria for PTSD as defined by the DSM-V. According to Gershuny and Thayer (1999), many people experience some kind of traumatic incident that results in psychological distress. The 12-month prevalence of OCD is approximately 30% among people with PTSD, which is significantly higher than the prevalence rate for the general population (Badour, Bown, Adams, Bunaciu, & Feldner, 2012). Additionally, the prevalence of OCD after a traumatic incident (not necessarily resulting in PTSD) has been studied extensively and the results have been widely variable, ranging from 30% to 82% depending on the population, dimensions, and criterion (Cromer, Schmidt, & Murphy, 2006; Fontenelle et al., 2012; Gershuny et al., 2008). Despite the inconclusive evidence for specific prevalence rates of trauma-induced OCD, the information does suggest that it should be considered and understood conceptually prior to working with clients with OCD with a history of traumatic experiences.

### 1.2. Understanding obsessive–compulsive disorder and trauma-related distress

OCD is a neuropsychological disorder characterized by intrusive and uncontrollable obsessions and compulsions that cause significant distress to the individual (APA, 2013). Obsessions are thoughts, urges, or images that the individuals experience as unwelcome and invasive. These obsessions cause discomfort to the individual with OCD by being anxiety provoking, guilt inducing (APA, 2013), and/or disgust-laden (Adams, Willems, & Bridges, 2011) depending on the nature of the obsession. The individual with OCD will actively try to avoid, subdue, or neutralize the obsession by engaging in avoidant behaviour or compulsions (APA, 2013). OCD is a highly individualized disorder. Obsessions may seem extremely illogical, counterintuitive, and disconnected, and yet themes of obsessions have been found across the population. Craighead, Miklowitz, and Craighead (2013) summarize the obsessional themes into the following categories: “(a) contamination; (b) guilt and responsibility for harm (to self or others); (c) uncertainty; (d) taboo thoughts about sex, violence, and blasphemy; and (e) the need for order and symmetry” (p. 81).

With OCD, the compulsions that follow obsessional thoughts, images, or urges play the specific role of decreasing discomfort for the client, but do not appear to be logically connected to the obsession (APA, 2013). For example, if an individual with OCD experiences an obsessional thought, such as “my family and friends are going to get hurt”, he or she may neutralize this obsession by feeling the compulsive need to turn the light switch on and off eight times before leaving the house. When this compulsion reduces the anxiety the client feels, the obsessional thought may then morph into the thought that “my family and friends will get hurt if I don't turn the light switch on and off eight times”. The majority of clients recognize that their obsessions and compulsions are unreasonable; nevertheless, there is a small portion of clients (approximately 4%) who have “absent insight/delusional beliefs” about their obsessions and compulsions (APA, 2013, p. 237).

Trauma-related distress has been defined as psychological distress as a direct result of experiencing a stressful event (APA, 2013). The psychological distress typically manifests as intrusive thoughts (e.g. flashbacks, nightmares, and hypervigilance), which are typically about the traumatic event experienced. The symptoms that an individual may experience are variable and situationally dependent. At times, the symptoms are clearly anxiety or fear oriented. At other times, the clinical picture includes depressive symptoms, anger and aggression, or dissociation. Any combination of these symptoms may be present after the exposure to an aversive or distressing event (APA, 2013). Conceptually, the overlap between OCD and trauma-related distress can be found in the way the individual thinks about and reacts to the intrusive thoughts inherent in the two situations.

Several theories of understanding OCD have been suggested over the years. The author will draw on PTSD research for understanding various models of trauma-related distress.

#### 1.2.1. Cognitive-behavioural models of OCD

Psychological models suggest that OCD develops out of a unique relationship between an individual's psychological functioning and his/her environment. Cognitive-behavioural models are the most widely accepted theories in explaining and understanding OCD (Craighead et al., 2013). Beck theorized in 1976 that dysfunctional adaptation is not a result of specific events, but rather an individual's inability to process and make sense of a said event (as cited in Craighead et al., 2013). Intrusive thoughts are experienced by roughly 80–90% of the general population (Briggs & Price, 2009). Despite the common occurrence of intrusive thoughts, the majority of people do not develop OCD. Normal intrusive thoughts transition into clinical obsessions when the individual takes on personal significance or responsibility for the thought. For example, an individual on vacation may experience the intrusive thought that someone is breaking into his/her home while he/she is away. The majority of the population would dismiss this thought as being insignificant and would relatively quickly assume that he/she has locked the door and all is well. A small portion of the population, however, would not be able to dismiss this thought and it may turn into a clinical obsession. The individual would then take on personal responsibility for events related to the thought (i.e. “if I think about it, it is sure to happen” or “I must take extra precaution to ensure it does not happen”). These thoughts would then become so intrusive that the individual would need to complete some action (i.e. refusing to go on holidays, replacing the negative thought with a positive thought, or checking that the door is locked a certain number of times before leaving) in order to reduce the distress he/she is feeling (Craighead et al., 2013).

Salkovskis, in 1985, was one of the first to develop a comprehensive cognitive-behavioural understanding of OCD. Previously, the most widely accepted theory was a behavioural model, which ignored the obvious link between cognition and psychopathology. Salkovskis (1985) combined the largely accepted behavioural theory with the relatively new cognitive theory. He believed that by combining behavioural and cognitive theories and treatments, clinicians would be able to use new approaches to intervene on treatment-resistant OCD. According to Salkovskis (1985), each person encounters potentially triggering stimuli at many points throughout any given day. Individuals who struggle with obsessional thinking, however, will actively avoid encountering potentially triggering stimuli. Salkovskis (1985) defines these intrusive thoughts as inherently ego dystonic (“the content is inconsistent with the individual's belief system, and is perceived as objectively irrational”, p. 578), and thus, the individual's reaction is determined by how impacting the intrusive thought is for the individual person. When the intrusive thought is viewed as being important and the individual places meaning on it, the person's belief system is shifted. The person takes on ownership, responsibility, or blame for the intrusive thoughts, which become automatic thoughts that are ego syntonic and result in affective disturbances (Salkovskis, 1985).

In Salkovskis’ (1985) model, next the individual may engage in neutralizing behaviour in order to reduce distress, which, if successful, reinforces the neutralizing behaviour. Even if the neutralizing behaviour does not reduce the anxiety, the unwanted event may not happen. For many, this is also a strong reinforcer and may also encourage more neutralizing behaviour in the future. If neither of the previous scenarios takes place, the neutralizing behaviour may become a powerful and unavoidable trigger in and of itself. Admittedly, Salkovskis (1985) identified a few challenges associated with his cognitive-behavioural model of OCD. Nevertheless, his theory created the groundwork for more advancement of the cognitive-behavioural understanding of OCD.

In 1997, Rachman proposed a cognitive theory of obsessions, which was developed out of the aforementioned cognitive theory of OCD from Salkovskis (1985). In this theory, Rachman (1997) suggests, “obsessions are caused by catastrophic misinterpretations of the significance of one's intrusive thoughts” (p. 793). As noted previously, most people experience intrusive thoughts, but Rachman (1997) identifies some differences between typically intrusive thoughts and atypical obsessional thoughts. Obsessions last longer, are more intense, more persistent, cause more distress, and create more lasting impact on the individual (Rachman, 1997, p. 793) and yet the content of typical and atypical intrusive thoughts are quite similar. Additionally, Rachman (1997) identified the key element that differentiates typical obsessions with problematic obsessions; namely, meaning. The meaning that an individual places on an intrusive thought, whether it be interpreting these thoughts as being “very important, personally significant, revealing, threatening, or catastrophic” (Rachman, 1997, p. 794), can shift a universally experienced and dismissed thought to an unavoidable obsession.

Rachman's (1997) theory also suggests obsessions are more likely to occur when an individual is exposed to stressful situations and that external cues often trigger obsessional thoughts. The more stressful the external cues, the greater the frequency of intrusive/obsessional thoughts, the greater the distress the individual will likely feel (Rachman, 2002). These stressful situations may be traumatic and/or aversive, which may provide evidence for the link between trauma and OCD. In 2002, Rachman suggested a similar theory for compulsions (specifically, compulsive checking). He suggested that compulsions occur when an individual believes he/she has a special responsibility to prevent unwanted events from occurring. Again, this theory can be applied to an understanding of trauma-related distress. If an individual feels responsible to prevent the traumatic event from reoccurring, he/she may respond with compulsions (as in OCD) or hypervigilance (in PTSD) or some other attempt to neutralize the anxiety experienced (Rachman, 1998).

#### 1.2.2. Cognitive-behavioural models of PTSD

Traumatic events result in primarily psychological symptoms; namely, “repeated and unwanted re-experiencing of the event, hyperarousal, emotional numbing, and avoidance of stimuli (including thoughts) which could serve as reminders for the event” (Ehlers & Clark, 2000, p. 319). As with OCD, many people experience at least some of these symptoms at some point in their lives. Most people's symptoms dissipate after a few months and no longer cause distress. There is a subgroup of the population, however, who experience these symptoms for many years after the event. As will be noted, cognitive-behavioural models of PTSD contain many similarities with cognitive-behavioural models of OCD.

As with OCD, PTSD problems begin with the individuals' thought appraisal. An individual is required to interpret and/or make meaning of the traumatic event and/or the thoughts immediately following the traumatic event (Ehlers & Clark, 2000). The individual interprets the events or event-related thoughts in a way that produces an immediate sense of threat or danger. As with OCD while the individual interprets intrusive thoughts as being personally significant and/or meaningful, individuals with PTSD interpret their intrusive thoughts about the traumatic event as being current, real, and personally significant. According to Ehlers and Clark (2000), this negative appraisal leads to an external threat (e.g. the world is unsafe) or, often, an internal threat (e.g. I am incapable of achieving the things I want in life). Furthermore, individuals can interpret events and event-related thoughts in a number of different ways: (1) they may interpret the threat as being more common by overgeneralizing the probability of threat reoccurrence; (2) they may interpret their emotional reactions as being revealing about who they are as a person (this can be seen in OCD, as well); and (3) they may interpret the event or event-related thoughts as having catastrophic consequences (again, similar to OCD); etc. (Ehlers & Clark, 2000).

Another similarity between cognitive-behavioural models of OCD and PTSD is the effort to neutralize the negative consequences of the misinterpretation of intrusive thoughts. The neutralization strategies used in PTSD range from thought suppression to safety behaviours, to isolation, etc. (Ehlers & Clark, 2000). The neutralizing behaviours anticipated to manage the distress experienced by the individual are dysfunctional for three reasons: (1) resulting in PTSD symptoms; (2) inhibiting change in negative interpretations of the trauma and/or trauma-related thoughts; and (3) inhibiting change in the nature of the trauma memory (Ehlers & Clark, 2000, p. 328).

The cognitive-behavioural model of understanding OCD has been combined with a trauma-response model of OCD to better understand the impact of traumatic experiences on the onset, development, maintenance, and treatment of OCD.

## 2. OCD and trauma

A traumatic event, as defined in the DSM-V (APA, 2013), is the “exposure to actual or threatened death, serious injury, or sexual violence … ” (p. 271). The author is suggesting a more liberal definition of trauma. Perhaps labelling it as “adverse experiences” or “stressful life events” (Briggs & Price, 2009; Fontenelle et al., 2012; McLaren & Crowe, 2003; Real et al., 2011; Rosso, Albert, Asinari, Bogetto, & Maina, 2012) would be more appropriate. Regardless, the author defines traumatic event as any event that causes the individual physical, emotional, or psychological distress. Thus, these events may include but are not limited to interpersonal conflict, loss of personal property, victimization, criticism or ridicule, illness, loss of trust, death of a loved one, war, natural disasters, car accidents, and/or divorce or separation from loved ones. Essentially, any event can be considered traumatic if the individual experiences it as such. By adopting this liberal definition of trauma, the author is better able to value the impact of critical life events on the individual. More specifically, the author is able to synthesize the current literature and research defining the impact of life events on the onset, development, maintenance, and treatment of OCD.

### 2.1. Developmental factors linking trauma and OCD

The developmental factors of a client presented with OCD are worth considering. Although there seems to be a gap in the literature regarding neurodevelopmental markers of the onset of OCD, other areas of development have been researched. As a client develops and creates ways of functioning in the world, tendencies towards maladaptive functioning and/or cognitive distortions may be present. By recognizing the possible developmental factors that may contribute to the manifestation of OCD, the clinician can be better equipped to provide adequate services to the individual. Briggs and Price (2009) found that a predisposition towards anxiety and depression tended to enhance the link between traumatic childhood experiences and OCD symptoms. Traumatic childhood experiences have an aversive impact on most children, not all children however respond to these experiences in the same way. Briggs and Price (2009) conclude that children, who have a tendency to be more anxious and/or depressed before the traumatic experience, are more likely to respond to the development of OCD.

Along the same lines, the occurrence of trait-anxiety within the family structure appears to be related to the sensitivity towards both traumatic events and the development of OCD (Huppert et al., 2005). Trait-anxiety is both taught and genetically passed on to children within the family unit. Once the individual has reached his/her anxiety threshold and OCD symptoms start to emerge, the severity of symptomatology appears to be related to the individual's level of distress tolerance, which is often modelled by caregivers. If caregivers are unable to teach and model adequate anxiety coping and distress tolerance skills, children are left to learn coping skills themselves and thus a personal responsibility for control of possible negative outcomes is established. This may develop as maladaptive coping techniques such as obsessions and compulsions as a way to manage the distress felt about situations that seem uncontrollable.

### 2.2. Possible links between OCD and PTSD

Recent research has suggested that OCD and PTSD are, in fact, two disorders on the same continuum (Gershuny, Baer, Radomsky, Wilson, & Jenike, 2003). Gershuny et al. (2003) suggest that there is a tremendous overlap between the symptomatology of both OCD and PTSD. Both are characterized by recurrent and intrusive thoughts that are experienced as anxiety/fear inducing. They discovered that as PTSD symptoms reduce, OCD symptoms increase, and as OCD symptoms are treated, PTSD symptoms take over. They argue that OCD symptoms do not appear to “replace” the PTSD symptoms, but rather OCD symptoms are used to cope with, reduce, and avoid the trauma-related symptoms and memories. Furthermore, the link between OCD and PTSD has been evidenced by a significant number of researchers (Badour et al., 2012; Huppert et al., 2005; Lafleur et al., 2011; Nacasch, Fostick, & Zohar, 2011; de Silva & Marks, 2001).

### 2.3. Post-traumatic OCD

Several theories of post-traumatic OCD have been suggested. Badour et al. (2012) suggest that the role of “disgust” in both OCD and PTSD is significant and worthy of further research. Those who experience traumatic victimization (sexual/physical/criminal assault) experience intense feelings of disgust. Feelings of disgust can lead to both PTSD (other-focused disgust) and a contamination-based OCD (self-focused disgust). These feelings lead to a need to remove oneself from sources of contamination and may result in hand washing, showering, and avoidance rituals. The intrusive recollections and thoughts, that are a result of the traumatic event, become generalized to other life experience (Sasson et al., 2004). They no longer appear to be conceptually associated with the traumatic event, but rather take on the typical patterns of obsessional thoughts found in OCD. Nevertheless, they can be traced back to the original traumatic event with time.

Rachman's (2010) theory of betrayal and contamination preoccupation adds additional evidence for the link between traumatic experiences and OCD. Although betrayal and physical contamination are not conceptually similar, sufficient evidence has been found that betrayal is relevant to anxiety disorders, OCD, and PTSD-like symptoms (Rachman, 2010, p. 304). Previously, clinicians have been focused on the traumatic event of PTSD and the compulsive behaviours and intrusive thoughts/obsessions of OCD, but Rachman (2010) argues that most patients can identify a critical betrayal event significant to the development of their disorder. As noted above, the current definition of trauma allows for the experience of personal betrayal to be considered even if it is not life threatening in nature. “Betrayal is a sense of being harmed by the intentional actions, or omissions, of a person who was assumed to be a trusted and loyal friend, relative, partner, colleague, or companion” (Rachman, 2010, p. 304). The five kinds of betrayal identified by Rachman (2010) are: “harmful disclosures of confidential information, disloyalty, infidelity, dishonesty, and failures to offer expected assistance during significant times of need” (p. 305). Rachman (2010) provides evidence that betrayal can lead to both OCD and PTSD-like symptoms. Given this overlap, one may deduce that OCD and PTSD are conceptually related.

Briggs and Price posited another theory of post-traumatic OCD in 2009. They suggested that early-life experiences created schemas and assumptions about the world. If these early experiences are seen as adverse and traumatic, children develop beliefs about personal responsibility. These children then inaccurately interpret intrusive thoughts as being the cause of negative events. For example, a child interpreting his/her negative thoughts about a parent as causing the car accident, or a child's belief that wishing his/her parents would stop arguing has caused his/her parents' divorce. An excessive number of negative events and propensity towards experiencing these events as being more impactful, may lead to the development of OCD (Gothelf, Aharonovsky, Horesh, Carty, & Apter, 2004). This theory also suggests the use of operant conditioning in the reinforcement of ritualistic behaviours to reduce anxiety. When an individual takes on personal responsibility for an intrusive thought, reacts by engaging in a compulsive behaviour, and then observes that something negative did not occur, he/she is reinforced to believe that the action is what prevented the negative event (Briggs & Price, 2009). Essentially, the development of OCD serves a protective function for those who have experienced traumatic events. The OCD obsessions and compulsions prevent the client from being further traumatized and thus help to reinforce the development of OCD (Fontenelle et al., 2007).

Finally, Lafleur et al. (2011) suggest that traumatic events may not cause OCD, but rather mediate the link between the environmental-genetic expression of OCD. In other words, the necessary environmental and genetic factors need to be present in order for a traumatic experience to trigger the onset of OCD. Therefore, the report of traumatic experiences in children with OCD may be over-represented because the child may have developed OCD later in life without the impact of the traumatic event.

### 2.4. Controversy

As the above theoretical review evidences, there has been debate among scholars as to the importance of traumatic incidences in understanding OCD. Most scholars, however, are aware of and can appreciate the presence of traumatic recall and the comorbidity of OCD with PTSD. Despite the lack of clear evidence, it is ascertained that understanding a client's traumatic history should be considered when working with those experiencing OCD. This is especially true because many individuals with prevalent traumatic events appear to be more resistant to traditional OCD treatments. Having an understanding of trauma therapies, the impact of trauma, and possible risk factors is necessary.

## 3. Clinical examples

The following section includes two examples from the author's clinical experience. These examples demonstrate the clear link between a traumatic experience and the onset of OCD.

### 3.1. Mrs. H

Mrs. H was a client seeking supportive counselling for her husband's terminal illness. The client first sought counselling approximately one year after her husband's diagnosis of Stage 3 colon cancer. Mrs. H reported that one month before her husband's diagnosis, she had experienced a debilitating stroke that completely disrupted her quality of life. She reported having a difficult time coping with both her and her husband's health concerns. Mrs. H reported symptoms of hypervigilance, insomnia, nightmares, mood disturbance, and flashbacks to her stroke. The clinical picture clearly represented PTSD-like symptoms, which were quite fitting for her reported history. After approximately six sessions, Mrs. H reported a desire to share what she described as embarrassing information. Mrs. H indicated that she found herself responding to the anxiety of her PTSD-like symptoms by engaging in ritualistic behaviour. When she coughed she felt compelled to cough at least five times in order to prevent her from having another stroke. When she would take her husband to his oncology appointments (specifically when the doctor would be reporting test results), she compulsively counted monitor beeps in sets of five in order to yield positive test results. At Mrs. H's admission, the clinical picture shifted to one of OCD-like symptoms. The link between her symptoms and the trauma of her stroke and her husband's terminal illness was clear. This clinical example demonstrates evidence that the PTSD and OCD may be activated in a very similar way and may coexist in some clients.

### 3.2. Mr. L

Mr. L was a client seeking treatment for OCD that had been diagnosed by a psychiatrist approximately six months prior to the first therapy session. The themes of Mr. L's obsessions were contamination based, specifically the harm to oneself and the harm to others. His compulsions were excessive hand washing and showering, and refusal to ride in/drive a vehicle. After a brief history was discussed, it came to light that Mr. L's symptoms emerged shortly after the client had been in a traumatic car accident. Mr. L was acutely aware of the harm he could have caused and did cause to those involved in the accident. He became preoccupied with preventing harm to others and himself, so much so that he feared receiving germs and passing germs onto others. The onset of OCD was directly linked to the perceived immediate threat (as in Ehlers & Clark, 2000; Rachman, 1997) and the misrepresentation of intrusive thoughts. The trauma he experienced in the car accident changed the way he interpreted his own thoughts and the safety of his experiences.

## 4. Treatment

Effective treatment of trauma-related OCD is defined as the reduction in obsessional thoughts and compulsory rituals. In addition, clinicians hope to see an increase in the quality of life and less distress placed on the client. Given the theoretical understandings of OCD, there are two primary forms of OCD and trauma-related distress treatments that have been tested and deemed effective. These treatments are based on the cognitive-behavioural therapy (CBT) model of counselling (Craighead et al., 2013).

### 4.1. Exposure treatments

Exposure and response prevention (ERP) is designed to expose the client to anxiety provoking stimuli and preventing the client from engaging in the compulsory ritual he/she is wanting to engage in by practising self-soothing, calming techniques (Craighead et al., 2013). Overtime, the anxiety elicited begins to subside and “habituation” (p. 99) begins to occur more rapidly as exposures are conducted. The purpose of ERP is to change the meaning that the individual places on the intrusive/obsessional thoughts. By reframing the meaning of these thoughts, the individual is better equipped to objectively review his/her intrusive thoughts and respond in an adaptive way.

Craighead et al. (2013) outline several studies attesting to the efficacy of ERP in treating OCD. Foa and Kozak found as high as 83% positive response rates at post treatment and 76% positive response rates at follow up (average of 29 months after final treatment; as cited in Craighead et al., 2013). Additionally, clients who undergo ERP treatment tend to see between 50% and 70% symptom reduction, a substantial benefit to those with OCD. Foa et al. also found that ERP was more effective than “weight list, progressive muscle relaxation, anxiety management training, pill placebo, and pharmacotherapy … ” (as cited in Craighead et al., 2013, p. 101). Despite the positive evidence, Adams et al. (2011) found that contamination-based OCD, which was linked to a personal victimization, showed more resistance to exposure-type treatments than other forms of OCD. Given this outcome, one can conclude that the initial use of ERP to treat OCD is justified, but that when dealing with traumatic-onset OCD, other treatment methods may be necessary.

Exposure treatments have been widely used as a way of intervening with individuals with PTSD. Specifically, Prolonged exposure and eye movement desensitization and reprocessing are largely accepted as effective exposure treatments for trauma-related distress (Rothbaum, Astin, & Marsteller, 2005). Similar to exposure treatments for OCD, the fear response is activated by eliciting memories related to the traumatic event. The client is then asked to withstand the anxiety produced until the anxiety subsides (Rothbaum et al., 2005). The overlap between OCD exposure treatments and trauma-related distress exposure treatments is significant, which implies, once again, conceptual similarities between the two disorders.

Shavitt et al. (2010) hypothesized that the presence of traumatic incidences of PTSD would negatively impact the OCD treatment outcomes. They concluded that a history of trauma did not negatively impact the non-treatment-resistant client's OCD treatment outcomes, but it did impact the outcomes for treatment-resistant OCD. Shavitt et al. (2010) actually found that the OCD + PTSD group showed greater improvement depending on the specific symptomatology. They also concluded that contamination-based obsessions and compulsions were more frequent in those who had a history of trauma and/or PTSD. Given that Adams et al. (2011) already found that contamination-based OCD was more resistant to traditional forms of OCD treatment, perhaps one can assume that there is an indirect relationship between trauma and treatment-resistant OCD. Shavitt et al. (2010) conclude their research project with a number of unanswered questions, stating that many of the novel findings in their study cannot be explained by the scope of their project. This is further evidence that more research into the interplay between traumatic experiences and OCD is necessary.

### 4.2. Cognitive-behavioural treatment

Although ERP has been used as the treatment of choice for OCD, there are still many individuals who do not benefit from it (Whittal & McLean, 1999). Over the last decade, clinicians have actively attempted to develop new approaches for treating OCD. CBT has become a widely accepted treatment for both trauma-related distress and OCD.

According to Whittal and McLean (1999), the goal of CBT is to correct faulty appraisals of intrusive thoughts. The obsessive–compulsive symptoms are maintained when the individual is unable to reinterpret or consider alternative meanings for the intrusive thoughts. Through identification of obsessions and compulsions, as well as the awareness of the way the individual interprets the obsessions, individuals are taught to challenge their own interpretations and revaluate the threat of them (Foa, 2010). Additionally, the therapist will challenge the client to behaviourally oppose his/her obsessions and compulsions as evidence contradicting the intrusive thoughts (Foa, 2010). Ehlers and Clark (2000) suggested similar treatment implications for the cognitive-behavioural treatment of PTSD. The trauma memory and/or trauma-related thoughts need to be reinterpreted and integrated into a past-oriented mindset. Additionally, neutralizing behaviours need to be modified to inhibit the perpetuation of PTSD symptoms. Finally, maladaptive behaviours need to be challenged and prevented to enhance the reinterpretation of trauma-related thoughts (Ehlers & Clark, 2000).

Being as intrusive thoughts, and arguably obsessional thinking, are evident in both OCD and trauma-related distress, Rachman's (1997) cognitive theory of obsessions provides significant treatment implications. Essentially, treatment of trauma-related OCD must focus on reinterpreting misinterpreted intrusive thoughts. If the interpretation of the trauma-related intrusive thought is redirected, the obsession should discontinue. Education about intrusive thoughts, normalization of the intrusive thoughts, recollection of and recording of the experienced intrusive thoughts, and, finally, the construction of new, adaptive, and appropriate interpretations (through identification of situational exceptions, alternative interpretations, and disproving evidence) are important steps in the treatment process (Rachman, 1997, 1998).

As can be seen from the above treatment overview, similarities between exposure treatments and CBT treatments of OCD and trauma-related distress are substantial. As a result, it is evident that trauma-related OCD treatment can be approached in a similar manner. The conceptual similarities between OCD and trauma-related distress (i.e. betrayal, intrusive thoughts, maladaptive appraisals, and behavioural neutralizing responses) provide evidence for OCD and trauma being very similar in presentation, course, and treatment.

## 5. Conclusions

Many gaps in knowledge regarding the impact trauma has on OCD have been highlighted throughout this paper. Further research into neurodevelopmental predisposition, developmental factors, type and theme of trauma, and treatment of trauma-specific OCD is necessary. Despite the lack of empirical evidence, clinical conclusions can be drawn from the synthesis of the present research.

### 5.1. Clinical recommendations

Regardless of whether a trauma theory of psychopathology is adhered to, the evidence suggesting the impact of trauma on OCD is irrefutable. Because trauma can complicate the treatment of OCD and/or have practical implications about the specific course of treatment, an accurate evaluation of the traumatic event, themes, and impact is necessary (de Silva & Marks, 1999). Exposure therapies and CBT have been well established as effective treatments for trauma-related disorders (Craighead et al., 2013) and, as noted above, OCD. Clinically, goals of treating both OCD and PTSD are similar but the overall aim shifts slightly. In using these techniques to treat OCD, the clinician seeks to reduce the anxiety associated with the intrusive thoughts and teaches the client to understand and respond to the intrusive thoughts differently. In using these techniques to treat PTSD and trauma, the clinician, again, seeks to reduce the anxiety associated with the intrusive thoughts and teaches the client to re-contextualize the intrusive thoughts, integrating the traumatic memory into a more helpful elaboration of the event. Thus, the clinician has the opportunity to treat both significant concerns simultaneously or consecutively (de Silva & Marks, 1999) depending on his/her clinical judgement regarding the most pervasive mechanism in the client's dysfunction. By neglecting the treatment of the trauma-related difficulties, the client will likely be resistant to OCD treatment and/or simply replace OCD symptomatology with other maladaptive coping techniques to manage the distress caused by the traumatic recall. By gaining a better understanding of the physical, psychological, and emotional distress the individual experienced as a result of the trauma, the clinician may gain insight into the obsessions and compulsions of the client with OCD and thus be better equipped to direct the treatment to maximize efficacy and enhance the client's quality of life.
